# Psychosocial Factors Associated with Cognitive Function in Prostate Cancer Survivors on Hormonal Treatments: A Systematic Review

**DOI:** 10.1007/s11065-024-09639-1

**Published:** 2024-04-20

**Authors:** Lorna Pembroke, Kerry A. Sherman, Heather Francis, Haryana M. Dhillon, Howard Gurney, David Gillatt

**Affiliations:** 1 Lifespan Health and Wellbeing Research Centre, Macquarie University, Macquarie Park, NSW 2109 Australia; 2 School of Psychological Sciences, Faculty of Medicine, Health and Human Sciences, Macquarie University & Macquarie University Hospital, Macquarie Park, NSW 2109 Australia; 3 Centre for Medical Psychology and Evidence-based Decision-making, School of Psychology, Faculty of Science, University of Sydney, Camperdown, NSW 2006 Australia; 4 Psycho-Oncology Cooperative Research Group, School of Psychology, Faculty of Science, University of Sydney, Camperdown, NSW 2006 Australia; 5 Faculty of Medicine, Health and Health Sciences, Macquarie University Clinical Trials Unit (CTU), Macquarie University & Macquarie University Hospital, Macquarie Park, NSW 2109 Australia; 6 Faculty of Medicine, Health and Health Sciences, Macquarie, Macquarie University Urology Clinic, University & Macquarie University Hospital, Macquarie Park, NSW 2109 Australia

**Keywords:** Prostate cancer, Hormone therapy, Cancer-related cognitive impairment, Neuropsychological intervention/rehabilitation, Psychosocial functioning

## Abstract

Hormonal treatments (HT) for prostate cancer (e.g., androgen deprivation therapy) yield clinical and survival benefits, yet adverse cognitive changes may be a side effect. Since psychosocial factors are largely modifiable, interventions targeting these factors may help mitigate these adverse cognitive effects. This systematic review aimed to identify a range of psychosocial factors associated with cognitive function in individuals with prostate cancer undergoing HT and to determine whether these factors mitigate or exacerbate this effect. Applying PRISMA guidelines, a comprehensive search of relevant databases conducted in September 2023 using terms related to prostate cancer, hormone therapy, and cognitive outcomes was undertaken. The search yielded 694 unique abstracts, with 11 studies included for analysis examining the relationship between cognitive function and the following psychosocial factors: psychological distress, fatigue, insomnia, and coping processes. Findings were mixed with only two studies reporting significant associations between cognitive performance with fatigue and depression. Three studies that included measures of perceived cognitive function identified associations with depression, anxiety, fatigue, insomnia, illness threat appraisals, and coping styles. However, no studies found evidence for an association between self-reported and objective measures of cognitive functioning. Evidence regarding the association of interpersonal factors is lacking. Moreover, whether these factors mitigate or exacerbate the effect of HT on cognitive function still needs to be determined. Overall, the research exploring the association between psychosocial factors and cognitive function in prostate cancer survivors undergoing HT is still in its infancy. Further research is required to optimize the implementation of neuropsychological interventions for prostate cancer survivors.

**Supplementary Information:**

The online version contains supplementary material available at 10.1007/s11065-024-09639-1.

Prostate cancer is one of the most prevalent malignancies in men worldwide. Hormone therapy (HT) is an effective treatment for prostate cancer yielding clinical and survival benefits. Different types of HT aim to suppress testosterone-induced tumor growth through mechanisms including reducing androgen production by the testes (e.g., orchiectomy, luteinizing hormone-releasing hormone (LHRH) or gonadotropin-releasing hormone (GnRH) agonists), blocking androgen production throughout the body (e.g., CYP17 inhibitors), and/or blocking the actions of androgen on the body (also known as antiandrogens, androgen receptor blockers, or antagonists) (American Cancer Society, 2022). HT is often combined with other treatment modalities (radiotherapy, surgery, chemotherapy) and can be administered continuously or intermittently (as guided by serum prostate-specific antigen levels) (American Cancer Society, 2022).

Playing a role in sexual and reproductive function, androgen and its metabolites (e.g., testosterone, estrogens) also have neuroprotective effects in maintaining cognitive functioning as demonstrated in human and animal studies (Cai & Li, 2020). In prostate cancer research, a variety of measures and methods have been used to assess cognitive functioning including self-report/subjective measures, neuropsychological/objective tests, and diagnostic assessments (Treanor et al., 2017). On self- or informant-reported (e.g., by a family member) measures, between 25 and 50% of men on androgen depleting/interfering HT experience cognitive impairments (Jenkins et al., 2005; Reiss et al., 2022; Wu et al., 2013, 2016). Some studies using objective neuropsychological testing report cognitive decline over time following initiation of HT in various cognitive domains including memory, language/verbal skills, reasoning, learning, attention, executive functioning, processing speed, and visuospatial skills (Bussiere et al., 2005; Ceylan et al., 2019; Chao et al., 2013; Green et al., 2004; Gunlusoy et al., 2017; Jenkins et al., 2005; Salminen et al., 2004; Yang et al., 2015a, b). Furthermore, there is evidence for an association between the use of HT for prostate cancer and an increased risk of developing dementia (Hong et al., 2020; Jhan et al., 2017; Tae et al., 2019; Tully et al., 2021).

Yet, not all studies consistently demonstrate changes to cognitive functioning after undergoing HT (Kluger et al., 2020), suggesting factors other than HT alone may be implicated. The extant literature has primarily investigated the role of potential biological, medical, and sociodemographic factors underlying cognitive functioning in men receiving HT. For example, older age, lower education levels, medical comorbidities (e.g., vascular risk factors), and longer administration of HT have been linked with increased risk for these HT-related cognitive declines (Nead et al., 2017b; Plata-Bello et al., 2019; Tae et al., 2019; Tully et al., 2021). Inconsistencies in the magnitude of effects and rates of cognitive change may also be partially attributed to methodological characteristics of the research, such as heterogeneity in follow-up duration and definitions of cognitive dysfunction, sample characteristics (e.g., sociodemographic factors, type of hormone therapy), and the type of cognitive tests used (Kluger et al., 2020; Treanor et al., 2017).

In addition, cognitive changes documented using objective measures often do not correlate with self-reported changes (Hutchinson et al., 2012). Rather, perceived cognitive changes have been found to be more strongly associated with psychosocial factors (e.g., depression, anxiety, poor coping strategies; Cull et al., 1996; Henneghan et al., 2021; Hutchinson et al., 2012). Furthermore, it has been argued that self-report measures may be more reflective of the *functional impact* rather than cognitive ability (Costa & Fardell, 2019). Therefore, exploring both objective and self-reported cognitive changes and related psychosocial factors is important in understanding moderating factors that may mitigate or enhance an individual’s risk for adverse HT impacts.

Despite the rapidly growing demand for effective management of cancer-related cognitive impairment or dysfunction, there are presently no standard treatments (Fernandes et al., 2019). Moreover, as most prostate cancer diagnoses occur in men aged 65 years or older, research in oncology and gerontology highlights the complex interaction of biological, psychological, socio-environmental, cancer, and treatment-related factors in moderating cognitive function in an ageing population (Lange et al., 2014). However, little attention has been given to the role of psychosocial factors, which are typically more modifiable than sociodemographic factors (e.g., education and age).

Psychosocial factors, as defined by the National Cancer Institute (cancer.gov), encompass affective, social, mental/psychological, and spiritual functioning. Research demonstrates the association between negative affect, notably depression, and objective cognitive decline, while emotional support and self-efficacy have been associated with better cognitive performance, independent of educational background, overall health status, and other psychosocial factors (Zahodne et al., 2014). Additionally, increasing evidence demonstrates the benefits of maintaining friendships in later life to maintain cognitive function (Sharifian et al., 2020; Zahodne, 2021). Identifying specific psychosocial factors that modulate the impact of prostate cancer-related HT on cognitive functions may be a critical first step in developing targeted interventions to address cognitive difficulties. Given the increasing importance of managing cancer-related cognitive impairment in survivorship, identification of modifiable psychosocial factors that may be protective or risk factors for men with prostate cancer receiving HT is a priority.

As psychosocial factors are largely modifiable and important avenues for intervention in other chronic health conditions (Deter, 2012), this systematic review aimed to synthesize and critically analyze research in the context of prostate cancer to identify psychosocial factors that may mitigate or enhance the impact of HT on cognitive functioning.

## Methods

This systematic review adhered to the PRISMA (Preferred Reporting Items for Systematic Reviews and Meta-Analyses) guidelines (Moher et al., 2009). The details of the protocol were prospectively registered on the Open Science Framework (https://osf.io/8f37q/). A broad literature search using keywords related to hormone therapy, prostate cancer, and cognitive dysfunction (Table 1) was performed (28 September 2023) using the following databases: MEDLINE/Ovid, PsychINFO, PubMed, EMBASE, CINAHL, and Web of Knowledge/Science. Reference lists of identified publications were also examined for relevant papers. Inclusion criteria were having (i) a prostate cancer survivor sample receiving hormonal-based treatments; (ii) at least one objective measure of cognition (i.e., not self-report); (iii) measure/s of psychosocial functioning; and (iv) an analysis of the relationship between cognitive outcomes and psychosocial factors. For the purposes of this review, psychosocial factors were defined as factors related to mental, emotional, social, and spiritual functioning, encompassing feelings, moods, beliefs, ways of coping, and interpersonal relations (National Cancer Institute; cancer.gov). Fatigue was included as a psychosocial factor given its affective/emotional aspect in cancer-related experiences (Campbell et al., 2022). Visual analogue scales to measure psychosocial factors were also included. Given the multidimensionality of many quality of life (QoL) measures, only related subscales (e.g., social and emotional functioning) were analyzed for the purposes of the review rather than QoL total scores. Papers reporting psychosocial factors only for study inclusion criteria purposes (i.e., meeting cut-offs to participate in the study) were excluded from the review. Non-English language papers, animal studies, reviews, meta-analyses, case studies/reports, retrospective/population database studies, qualitative studies, preprint literature, conference abstracts, and poster presentations were excluded. Papers that included orchidectomy (i.e., surgical removal of the testicles) in their definition of HT were not excluded.

**Table 1:** Search terms for the systematic review

Population	Intervention	Outcome	
Prostate cancer/neoplasm/tumor/carcinoma/oncolog*/malignan*	Androgen deprivation therapy (HT) Androgen suppression therapy Antihormone therapy Antiandrogens: • Flutamide • Nilutamide • Enzalutamide • Bicalutamide Antineoplastic Androgen blockage Androgen antagonist Gonadotropin-releasing hormone Luteinizing hormone-releasing hormone (LHRH) agonists: • Leuprolide • Goserelin • Triptorelin • Histrelin • Chemical castration LHRH antagonist: Degarelix CYP17 inhibitor: abiraterone Antifungal: ketoconazole	Cogniti* Neuropsychol* Neurocognit*	impair* deficit disturb* impact disorder outcome

Eligibility assessment was performed by two reviewers (LH, KS), and disagreements between reviewers were resolved by discussion until consensus was achieved. A data-charting form was jointly developed by the reviewers, extracting information on article characteristics (e.g., country of origin, year of publication), data reported on cognitive outcomes, and psychosocial factors. A modified version of the QUADAS-2 (Quality Assessment of Diagnostic Accuracy Studies; Whiting et al., 2011) tool was used to assess the risk of bias and applicability of the included studies for the review. The signalling questions for each domain have been adapted for the purposes of this review, as recommended by the QUADAS-2 guidelines, and are depicted in the Supplementary Material.

Adherence to the International Cognition and Cancer Task Force (ICCTF) recommendations to harmonize studies of cognitive function in survivors with cancer was also examined (Wefel et al., 2011). Post-hoc power analyses were conducted using G*power (Faul et al., 2007); a power of 0.8 or greater to detect a medium effect size was deemed adequate to detect mild-to-moderate cognitive impairments typically reported in research examining non-central nervous system (CNS) cancer-related cognitive impairments (Bezeau & Graves, 2001; Lange et al., 2019).

## Results

### Descriptive Statistics

**Fig. 1:**
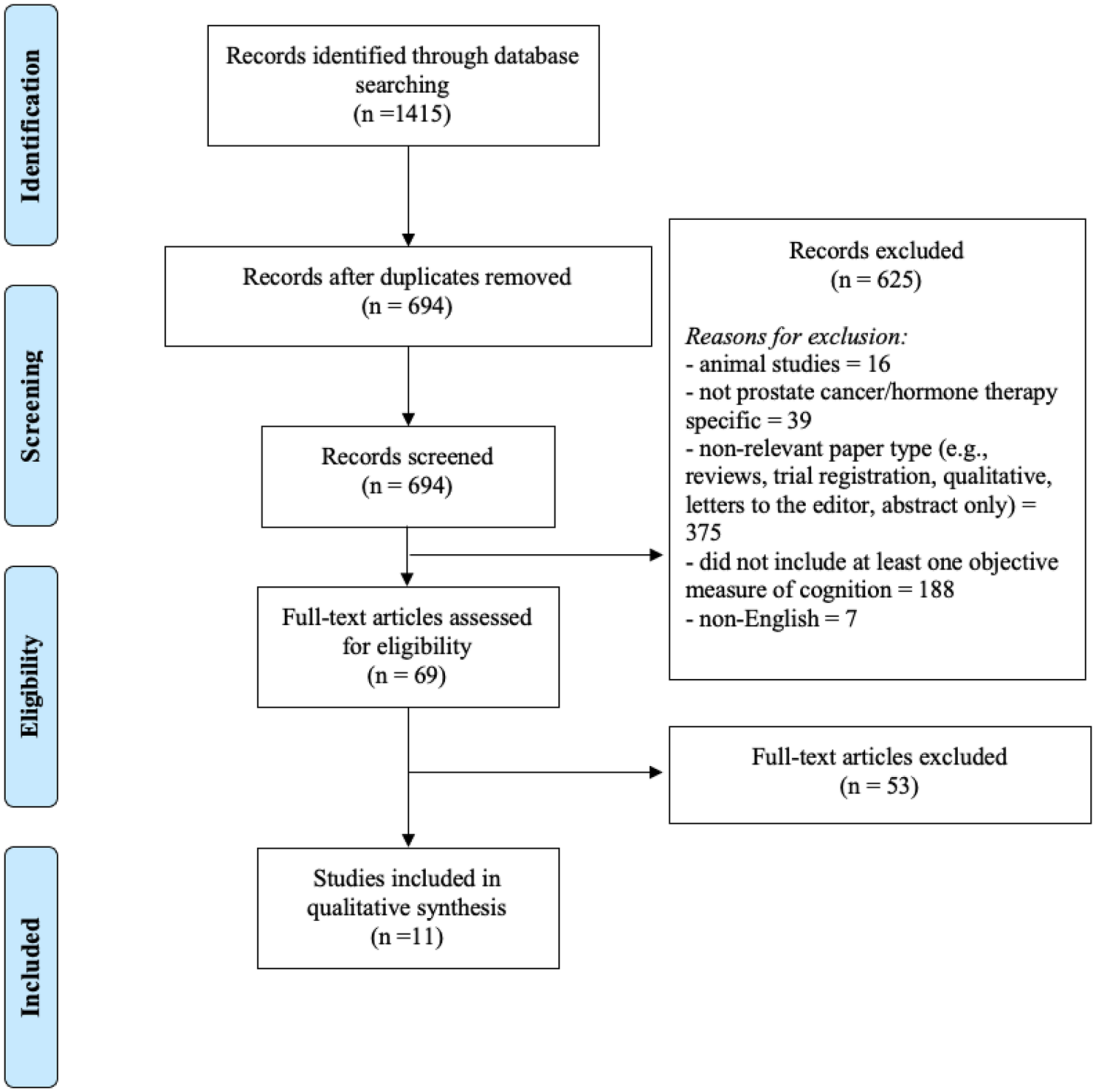
PRISMA flow diagram of the screening process

The search yielded 1415 papers with 694 unique abstracts screened after duplicates were removed. After excluding 625 abstracts, 69 studies underwent full-text screening, identifying 11 studies that examined the association between cognitive and psychosocial functioning (see Table 2), which are explored in detail by this review. Figure 1 presents a flow diagram of the screening process.

**Table 2:** Details of studies included in review

**Author/s (year), location**	**Title**	**Study design (assessment timepoints)**	**Participants**	**Hormone therapy details**	**Cognitive measures**	**Psychosocial measures**	**Relevant findings**
Papers with both *objective* and *self-report* measures of cognitive functioning							
Green et al. (2002b), Australia	Coping and Health-Related Quality of Life In Men With Prostate Cancer Randomly Assigned To Hormonal Medication Or Close Monitoring	Randomized controlled trial (baseline, 6 months)	*n* = 65 men with non-localized PC randomly assigned to receive LHRH analogues, a steroidal antiandrogen or close clinical monitoring *n* = 16 community volunteers matched for age and general health	Continuous LHRH: leuprorelin and goserelin Steroidal antiandrogen: cyproterone acetate	Processing speed: TMT-A Attention: concentration index of the WMS-R Working memory: WAIS DS Visuospatial: ROCFT Memory: WMS-R verbal subtests, visual subtests; AVLT Executive functioning: TMT-B; COWAT; Stroop test Self-report: Cognitive functioning subscale of EORTC-QLQ-C30	Psychological distress: DASS-21 Coping: items developed to measure threat and self-efficacy appraisals; COPE Others: Satisfaction with Life Scale; Subscales of EORTC-QLQ-C30	-ADT was associated with decreases in self-reported social/role and subjective cognitive functions -Lower threat appraisals at baseline were associated with higher self-reported existential satisfaction, social/role and subjective cognitive functions - Higher use of either emotion- or problem- focused coping was associated with higher emotional distress at baseline, and with decreased social/role and subjective cognitive function at 6 months
Marzouk et al. (2018), Canada	Impact of Androgen Deprivation Therapy on Self-Reported Cognitive Function in Men with Prostate Cancer	Prospective, observational, comparative study (baseline, 6, 12 months)	*n* = 81 ADT-treated men with non-metastatic PC *n* = 84 controls with PC not receiving ADT *n* = 85 healthy controls all age- and education-matched	Continuous ADT	Processing speed: TMT-A Working memory: WAIS DS, Spatial Span; Spatial Working Memory Task Visuospatial: JLO, Card Rotations Memory: CVLT; BVMT; Conditional Associative Learning Test Executive functioning: TMT-B; COWAT; DKEFS Color-Word Interference Test Self-report: FACT-Cog	Psychological distress: 15-item GDS Fatigue: Vitality subscale of 36-Item Short Form Survey	-Mood and fatigue correlated with changes in self-reported cognitive function - ADT was not associated with self-reported cognitive function changes in men with non-metastatic prostate cancer - Weak relationship between self-report and objective cognitive measures
Tulk et al. (2023), Canada	Androgen Deprivation Therapy and Radiation for Prostate Cancer— Cognitive Impairment, Sleep, Symptom Burden: a Prospective Study	Prospective, observational study (baseline, 12 months)	*n* = 24 men newly diagnosed with PC on ADT + RT	Gonadotropin-releasing hormone agonist	Working memory: WAIS-IV Letter Number Sequencing Memory: HVLT Executive functioning: COWAT Self-report: FACT-Cog	Psychological distress: HADS Fatigue: Multidimensional Fatigue Symptom Inventory- Short Form	- Objective cognitive decline did not correlate with changes in any self-reported outcome measures - Declines in perceived cognition functioning was associated with higher anxiety, fatigue and symptoms of insomnia
Papers with only * objective* measures of cognitive functioning							
Almeida et al. (2004), Australia	One Year Follow-up Study of the Association Between Chemical Castration, Sex Hormones, Beta-amyloid, Memory and Depression in Men	Prospective, observational study (pre-baseline, baseline, 4, 12, 24, 36, 42, 48, and 54 weeks)	*n* = 40 PC survivors	Intermittent ADT with leuprolide and flutamide	General cognition: CAMCOG Memory: WMS-III Word Lists, VPA, VR Visuospatial: WAIS-III BD	Psychological distress: BDI and BAI	- Significant improvements seen on CAMCOG, verbal memory and percentage retention on VR after ADT discontinuation - Changes in BDI and BAI could not explain improved cognitive performance
Bussiere et al. (2005), USA	Androgen Deprivation Impairs Memory in Older Men	Cross-sectional study	*n* = 14 PC survivors (non-metastatic and metastatic) *n* = 16 age-matched healthy men	Leuprolide acetate (*n* = 12) and orchiectomy (*n* = 2)	Computerized verbal memory tasks examining encoding, retention interval, and recognition	Psychological distress and fatigue: POMS	- Men on ADT had a specific impairment of retention but normal encoding and retrieval processes on a word list-learning task - ADT men reported greater fatigue levels than controls. When fatigue was added as a covariate, it either moderated memory performance or decreased the power to detect differences
Cherrier et al. (2009), USA	Cognitive and Mood Changes in Men Undergoing Intermittent Combined Androgen Blockade for Non-Metastatic Prostate Cancer	Prospective, observational, comparative study (baseline, 3, 9, and 12 months)	*n* = 20 hormone naïve, eugonadal PC survivors without evidence of metastases and with a rising PSA treated with ADT *n* = 20 controls without PC, matched for age and education	Intermittent ADT for 9 months, combined leuprolide and flutamide followed by an “off treatment” period	Working memory: Subject Ordered Pointing Test Visuospatial: WAIS BD; Vandenburg and Kuse Mental Rotation Test Memory: Puget Sound Route Learning Test; Moscovitch’s Proactive Interference test; WMS-R LM Executive functioning: Verbal fluency: “P” word generation; Stroop test	Psychological distress and fatigue: POMS, Visual analogue scale (VAS) measuring irritability, tension, depression, moodiness	-ADT survivors demonstrated a significant decline in spatial reasoning, spatial abilities and working memory, and increases in self-rated depression, tension, anxiety, fatigue and irritability during treatment compared with baseline - No significant interaction between mood and cognitive measures
Gonzalez et al. (2015), USA	Course and Predictors of Cognitive Function in Patients With Prostate Cancer Receiving Androgen-Deprivation Therapy: A Controlled Comparison	Prospective, observational, comparative study (baseline, 6, 12 months)	*n* = 58 PC survivors starting ADT (non-metastatic or asymptomatic metastatic) *n* = 84 PC survivors treated with prostatectomy only (age- and education-matched) *n* = 88 men without PC (age- and education-matched)	Not specified	Processing speed: Color Trails; SDMT Working memory: WMS-III DS, Spatial Span Memory: HVLT-R; WMS-III LMII; BVMT-R Executive functioning: Color Trails; COWAT Others: Timed Instrumental Activities of Daily Living Premorbid intelligence estimate: NART Full-Scale IQ	Psychological distress: Center for Epidemiologic Studies Depression Scale Fatigue: FSI	-ADT recipients demonstrated higher rates of impaired cognitive performance over time relative to all controls -Depressive symptoms, fatigue, and hot flash interference did not moderate the impact of ADT on impaired cognitive performance
Green et al. (2002a), Australia	Altered Cognitive Function in Men Treated for Prostate Cancer with Luteinizing Hormone-releasing Hormone Analogues and Cyproterone Acetate: a Randomized Controlled Trial	Randomized controlled trial (baseline, 6 months)	*N* = 82 men with extra-prostatic PC were randomly assigned to receive LHRH analogues, a steroidal antiandrogen or close clinical monitoring	Continuous LHRH: leuprorelin and goserelin Steroidal antiandrogen: cyproterone acetate	Processing speed: TMT-A Attention: concentration index of the WMS-R Working memory: WAIS DS Visuospatial: ROCFT Memory: WMS-R verbal subtests, visual subtests; AVLT Executive functioning: TMT-B; COWAT; Stroop test	Psychological distress: DASS-21	-Deficits across a range of tasks suggesting ADT may disrupt complex information processing rather than with memory specifically -Differential effects of goserelin, cyproterone, and leuprorelin on cognition -Cognitive changes not associated with mood changes
Papers with only * objective* measures of cognitive functioning—cognitive screening measure							
Araújo et al. (2022), Portugal	Androgen-deprivation Therapy and Cognitive Decline in the NEON-PC Prospective Study During the COVID-19 Pandemic	Prospective, observational, comparative study (baseline, 12 months)	*n* = 186 survivors on ADT (including adjunct RT, RP, and chemotherapy) *n* = 180 survivors on active surveillance, brachytherapy, RT, RP	Goserelin with or without bicalutamide/degarelix/abiraterone acetate and enzalutamide	MoCA	Psychological distress: HAM-D	- Cognitive decline was more frequent in the ADT group - Psychological distress was not associated with cognitive decline
Ceylan et al. (2019), Turkey	The Depressive Effects of Androgen Deprivation Therapy in Locally Advanced or Metastatic Prostate Cancer: a Comparative Study	Prospective, observational, comparative study (baseline, 6, 12 months)	*n* = 72 survivors with locally advanced or metastatic PC on ADT *n* = 72 (control) survivors who underwent radical prostatectomy without any additional treatment	Continuous ADT for 12 months	MoCA	Psychological distress: HAM-D	-Association between depression, deterioration of language, memory functions and attention at 6th and 12th months
Sanchez-Martínez et al. (2021), Spain	Analysis of Brain Functions in Men with Prostate Cancer under Androgen Deprivation Therapy: A One-Year Longitudinal Study	Prospective, observational study (6- and 12-month follow-up)	*n* = 33 PC survivors on ADT (non-metastatic and metastatic)	LHRH analogues	MMSE; The Brief Scale for Cognitive Evaluation	Psychological distress: GDS	-No association between ADT and cognitive deterioration -No differences in the cognitive performance between men with or without impaired sleep or depression

*ADT *Androgen Deprivation Therapy,* AVLT* Auditory Verbal Learning Test, *BAI* Beck Anxiety Inventory, *BD* block design, *BDI* Beck Depression Inventory, *BVMT-R* Brief Visuospatial Memory Test—Revised, *CAMCOG* Cambridge Examination for Mental Disorders of the Elderly—Cognitive Battery Revised, *COPE* Coping Orientation to Problems Experienced Inventory, *COWAT* Controlled Word Association Test, *CVLT* California Verbal Learning Test, *DASS-21* Depression Anxiety Stress Scales—21 items, *DKEFS* Delis–Kaplan Executive Function Scale, *DS* digit span, *EORTC-QLQ-C30* European Organization for the Research and Treatment of Cancer Core Quality of Life Questionnaire, *FACT-Cog* Functional Assessment of Cancer Therapy-Cognitive Function, *FSI* Fatigue Symptom Inventory, *GDS* Geriatric Depression Scale, *HADS* Hospital Anxiety and Depression Scale, *HAM-D* Hamilton Depression Rating Scale, *HVLT* Hopkins Verbal Learning Test, *JLO* Judgment of Line Orientation, *LHRH *Luteinizing Hormone-Releasing Hormone, *LM* Logical Memory, *MMSE *Mini-Mental State Examination*, MoCA* The Montreal Cognitive Assessment, *NART* National Adult Reading Test, *ROCFT/RCFT* Rey-Osterrieth Complex Figure Test, *RP* Radical Prostatectomy, *RT* Radiotherapy, *SDMT* Symbol Digit Modalities Test, *PC* Prostate Cancer, *POMS* Profile of Mood States, *TMT* Trail Making Test, *VPA* Verbal Paired Associates, *VR* Visual Reproductions, *WAIS* Wechsler Adult Intelligence Scale, *WMS* Wechsler Memory Scale

Of the 11 studies, there were two randomized controlled trials (RCT), eight prospective observational studies (five with comparison groups, three without), and one cross-sectional study. Total sample sizes ranged from 24 to 366 participants. Two studies recruited men with non-metastatic, localized prostate cancer; three recruited men with non-localized (i.e., locally advanced or metastatic) prostate cancer; and five recruited both men with localized and non-localized prostate cancer. Most studies employed a battery of neuropsychological tests that assessed multiple domains (attention, processing speed, working memory, visuospatial functioning, memory, executive functioning). Of these studies, three also included a measure of self-reported cognitive functioning. Three papers used only cognitive screening measures to assess cognition. A range of psychosocial measures were employed measuring health-related quality of life, psychological distress/emotional functioning, coping responses, self-efficacy, sleep disturbance, and fatigue.

### Quality Assessment and Risk of Bias

**Fig. 2:**
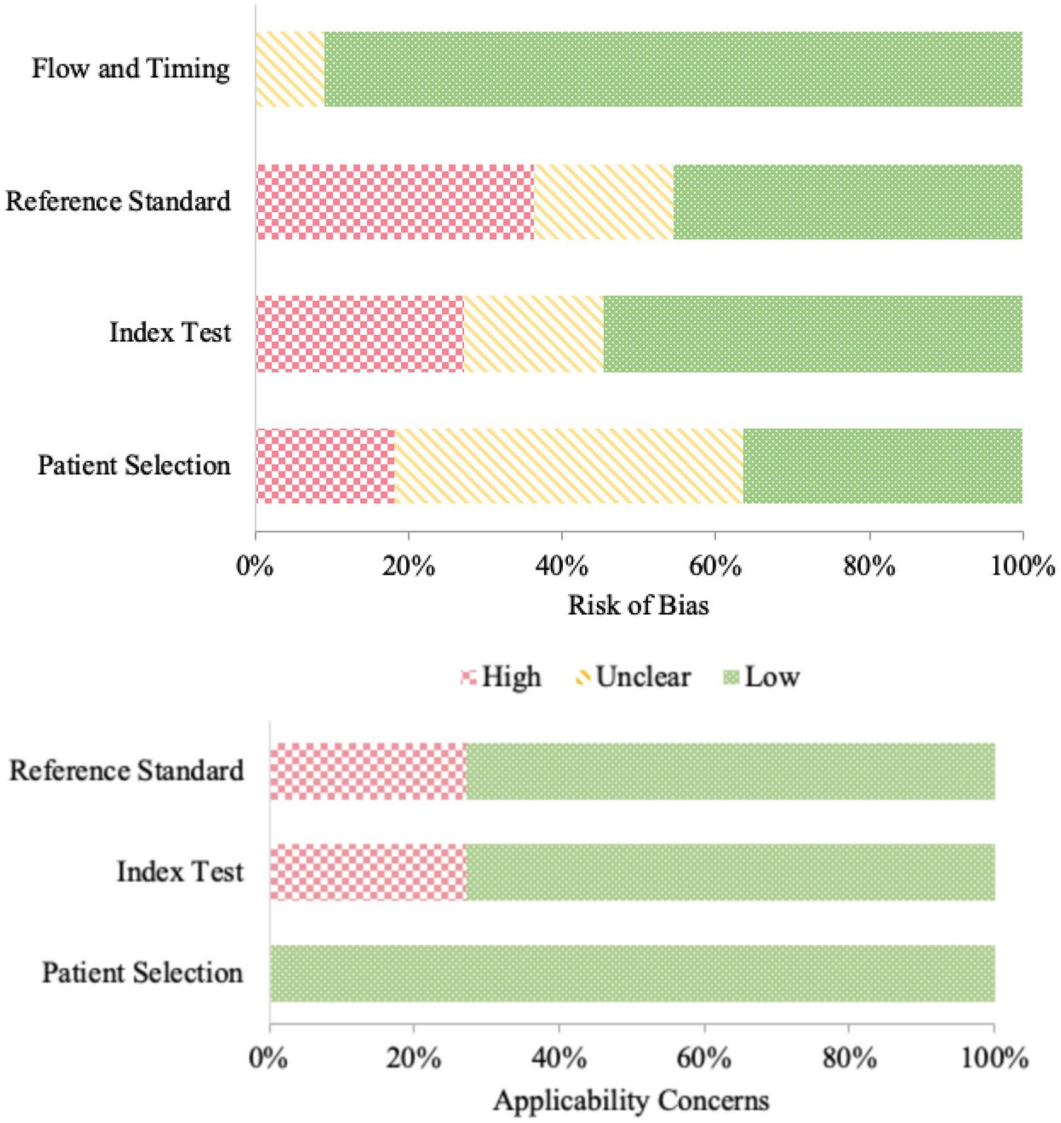
Proportion of studies with low, high, or unclear risk of bias and applicability concerns

The QUADAS-2 quality assessment and risk of bias evaluation are summarized in Table 3 with the overall results displayed graphically in Fig. 2. Regarding participant selection, four studies (36%) were judged as having a low risk of bias due to the use of random or consecutive sampling methods, and two studies (18%) not applying such methods were deemed as low risk of bias. Five studies (45%) did not clearly outline their sampling methods. Regarding the index tests (i.e., whether the conduct or interpretation could have introduced bias), six studies (55%) were judged as having a low risk of bias given the use of pre-specified criteria for cognitive impairment and appropriate neuropsychological measures; two of these studies had assessors blinded to the treatment condition. Three studies (27%) had a high risk of bias due to using only cognitive screening tools, which lack sensitivity to detect mild-to-moderate cognitive impairments. Regarding the reference standard (i.e., whether cognitive impairment criteria followed ICCTF recommendations), five studies (45%) had a low risk of bias, whereas four studies (36%) had a high risk of bias given the use of cognitive screening tools. The risk of bias was unclear for two studies (18%) given the use of comparison groups to assess differences in cognitive function rather than set criteria for cognitive impairment; the absence of prostate cancer controls may also introduce bias related to disease rather than treatment specific cognitive changes. In terms of flow and timing, almost all the studies (91%) were judged as having a low risk of bias, except one which was unclear given the limited explanation provided for dropouts. Regarding judgments of applicability, there was a low level of concern for most studies in most domains except the three studies (27%) using only cognitive screening tools. Even though cognitive screeners may not be sensitive enough to detect non-CNS cancer-related cognitive changes, these studies nevertheless explored the association between cognitive functioning and psychosocial factors and provided some relevant preliminary insights.

**Table 3:**
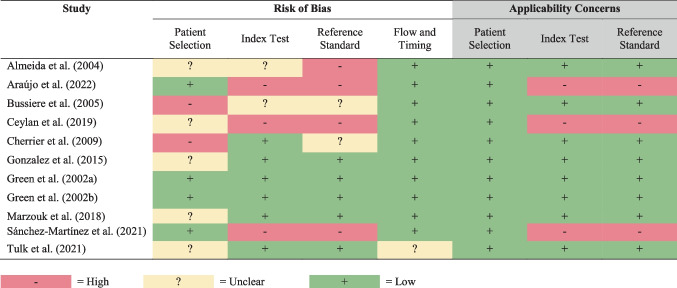
QUADAS-II risk of bias and applicability concerns summary table

Table 4 depicts the adherence of the studies with ICCTF recommendations and whether they were adequately powered. Three studies (27%) included both a prostate cancer and noncancer control group, five studies (45%) adhered to criteria for assessing cognitive impairments, and four (35%) followed the recommendations for neuropsychological testing. Moreover, seven studies (64%) conducted both baseline (pre-treatment) and follow-up assessments. Post-hoc analyses indicated six studies (55%) were adequately powered to detect clinically significant changes in cognitive functioning.

**Table 4:** Adherence to ICCTF recommendations and post-hoc power analysis

**Study**	**ICCTF recommendations**				**Other**	
	Inclusion of control/comparison groups^a^	Criteria for cognitive impairment^b^	Use of recommended measures^c^	Pre-treatment baseline assessment and follow-up		Adequately powered study
Almeida et al. (2004)	✘	✘	Partially met	✔		✔
Araújo et al. (2022)	Partially met	✘	✘	✔		✔
Bussiere et al. (2005)	Partially met	✘	✘	✘		✘
Ceylan et al. (2019)	Partially met	✘	✘	✔		✘
Cherrier et al. (2009)	Partially met	✘	Partially met	✔		✘
Gonzalez et al. (2015)	✔	✔	✔	✔		✔
Green et al. (2002a)	Partially met	✔	✔	✔		✔
Green et al. (2002b)	✔	✔	✔	✔		✔
Marzouk et al. (2018)	✔	✔	✔	Partially met		✔
Sanchez-Martínez et al. (2021)	✘	✘	✘	Partially met		✘
Tulk et al. (2023)	✘	✔	Partially met	✔		✘

✔= Recommendations met

✘ = Recommendations not met

*ICCTF* International Cognition and Cancer Task

^a^Inclusion of both a prostate cancer control and noncancer control groups

^b^Specification of cut-off points or definition of impairment using individual tests and overall battery

^c^Employed neuropsychological measures with adequate psychometric properties suitable for multinational application and alternate forms available. Measures should assess learning, memory, processing speed, and executive functioning, especially using the following tests: Hopkins Verbal Learning Test-Revised (HVLT-R), Trail Making Test (TMT), and the Controlled Oral Word Association Test (COWAT) of the Multilingual Aphasia Examination)

### Association Between Objective Cognitive Functioning and Psychosocial Factors

Most studies reported changes on both cognitive and psychosocial measures. Only two studies found a significant association between objective cognitive functioning and psychosocial measures.

#### Psychological Distress

All studies included a measure of psychological distress (e.g., depression). Only one study reported a significant association between depression and cognitive functioning. Ceylan et al. (2019), using clinician-administered structured interviews to assess depression, found prostate cancer survivors on HT not only performed worse on cognitive testing but demonstrated greater depressive symptomology over time than prostate cancer survivors without HT. Moreover, prostate cancer survivors on HT diagnosed with depression performed especially poorly on measures of attention, language, and memory abilities at 6 and 12 months post commencement of HT, yielding large effect sizes (from Cohen’s *d* = 0.57 to 1.03, except for language abilities at 12 months *d* = 0.08). However, these results should be interpreted cautiously as the study used a cognitive screening measure (Montreal Cognitive Assessment) and was inadequately powered.

Most studies (75%) did not find an association between self-reported psychological distress or mood and cognitive function in survivors on HT. Evidence of cognitive decline on neuropsychological testing was not associated with self-reported mood in five studies (Araújo et al., 2022; Cherrier et al., 2009; Gonzalez et al., 2015; Green et al., 2002a; Green et al., 2002b; Tulk et al., 2023). Moreover, one study that found improvements in cognitive performance following discontinuation of HT also could not be explained by changes in depression or anxiety (Almeida et al., 2004). Another study using cognitive screening (Mini-Mental State Examination) found no evidence of cognitive decline nor an association with reported increased depressive symptomology over time (Sanchez-Martinez et al., 2021).

#### Fatigue

Four studies examined the association between fatigue and cognitive function. Fatigue did not moderate the impact of HT on neuropsychological test performance in three studies (Cherrier et al., 2009; Gonzalez et al., 2015, Tulk et al., 2023). One study found that when fatigue levels were included as a covariate in a comparison of men on HT and noncancer controls, differences in memory retention, along with the interaction effect, were no longer statistically significant (Bussiere et al., 2005). This suggests fatigue either moderated memory performance or decreased the power to detect differences.

### Association Between Subjective Cognitive Functioning and Psychosocial Factors

The three studies including a measure of subjective cognitive functioning found significant associations with psychosocial factors. Marzouk et al. (2018) reported self-reported levels of depressive symptoms and fatigue were significant predictors of self-reported cognitive changes. Similarly, Tulk et al. (2023) found declines in perceived cognition functioning were associated with increased anxiety, fatigue, and symptoms of insomnia, even though both studies found no association between these psychosocial factors with objective measures of cognition.

A single study reported the relationship between coping processes (threat appraisals of illness and coping styles) and self-reported cognitive functioning (Green et al., 2002b). Lower threat appraisals (e.g., rating of how stressful the difficulties associated with cancer are) at baseline were associated with higher self-reported cognitive functions at baseline and after 6 months of HT. High use of coping behaviors (emotion- or problem-focused) was also associated with lower self-reported cognitive functioning at 6 months. The study provided limited information on the breakdown of coping behaviors employed (i.e., whether men engaged in more proactive/adaptive strategies).

### Association Between Subjective and Objective Cognitive Functioning

Of the three studies including both subjective and objective measures of cognitive functioning, only two analyzed the relationship between these types of measures. Marzouk et al. (2018) found changes in self-reported cognitive changes were weakly correlated with objective measures of cognition (i.e., with the maximum Spearman correlation coefficient being 0.14 for Judgement of Line Orientation and Spatial Span Backwards Task). Furthermore, Tulk et al. (2023) found that changes in cognitive performance did not significantly predict changes in self-reported cognition.

## Discussion

In this review, we aimed to synthesize and critically analyze published research exploring the association between psychosocial factors and cognitive function in men with prostate cancer receiving HT and whether these factors mitigate or exacerbate the effect of HT on cognitive function. Overall, few studies have specifically examined this association and possible moderation by psychosocial factors of HT effects on cognitive function. Most of the reviewed studies reported on declines in cognition and psychosocial factors (specifically increased levels of psychological distress and fatigue) in men undergoing HT. The evidence, however, for the association of these two factors was mixed. The few studies adhering to ICCTF recommendations did not find a significant association between psychosocial factors and cognitive function, whereas studies using self-report measures of cognitive functioning did (Green et al., 2002b; Marzouk et al., 2018, Tulk et al., 2023). No reviewed study identified an association between self-report and objective measures of cognitive functioning.

### Psychological Distress

Impairments in cognitive function are a well-established feature of clinically elevated depressive symptomology (Pan et al., 2019). However, this was observed in only one of the reviewed studies (Ceylan et al., 2019), which used clinician-administered structured interviews to assess mood disturbance. Most studies included self-report measures of psychological distress, whereby the association with cognitive performance appeared less pronounced or nonsignificant. Nevertheless, most reviewed studies observed increases in depressive symptomology over time, which is consistent with prior research reporting an association between HT and increased risk of depression (see Nead et al., 2017a, b for a meta-analysis). However, whether levels of depressive symptomology reached clinical thresholds in these studies was either not analyzed (Gonzalez et al., 2015; Cherrier et al., 2009) or observed (Green et al., 2002a, b). It is important to consider the potential of response biases obscuring the clinical picture. Men may underreport symptoms of depression (Sigmon et al., 2005), and not all measures are sensitive in capturing symptoms men present with or recognize (see Male Depression Risk Scale, Herreen et al., 2022; Oliffe et al., 2019). Overall, these findings highlight the value of structured clinical interviews and the use of gender-sensitive measures of psychological distress in prostate cancer populations, which may help elucidate its association with cognitive functioning.

### Fatigue and Insomnia

We found preliminary evidence for the association of fatigue and insomnia with perceived cognitive functioning (Marzouk et al., 2018; Tulk et al., 2023) rather than on objective tests. Problems with fatigue are prevalent among prostate cancer survivors on HT (Nelson et al., 2016), with as many as 43% reporting clinically significant levels (Storey et al., 2012). HTs increase the risk of insomnia for prostate cancer survivors, likely due to the increased presence of hot flashes and night sweats (Savard et al., 2013). Insomnia symptoms have been found to mediate the relationship between HT and self-reported cognitive functioning with the relationship between these factors being significantly moderated by fatigue and depression (Garland et al., 2021). Thus, interventions aimed to improve sleep, fatigue, and/or depression may indirectly improve perceptions of cognitive functioning.

While some research exists promoting exercise and diet interventions in managing fatigue in prostate cancer survivors (Baguley et al., 2017), whether these interventions improve cognitive outcomes is unknown. Fatigue management embedded within neuropsychological interventions has been associated with improvements in both self-report and objective cognitive functioning in cancer survivors (Green et al., 2018; Mihuta et al., 2018; Schuurs & Green, 2013). These studies, however, were mainly pilot in nature, not prostate cancer-specific, and the effect of fatigue management itself could not be isolated. Therefore, more research is required investigating, firstly, the extent to which fatigue impacts cognitive functioning in survivors receiving HT and, secondly, the benefits of fatigue management in improving cognitive outcomes.

### Coping Processes

In the extant literature, having informed expectations and understanding of cancer (i.e., illness representation) is important in improving overall adjustment to and coping with the disease (Richardson et al., 2017). Some evidence in noncancer populations suggests coping behaviors may moderate the effect of stress on cognitive function (Zhu et al., 2019), yet this research is still exploratory. A single study in our review found an association between poorer self-reported cognitive functioning and increased threat appraisals of illness and the use of coping behaviors in survivors receiving HT (Green et al., 2002b). However, limited information was provided on the influence of specific coping behaviors that may moderate cognitive function.

### Interpersonal Factors

Little is known about the relationship between interpersonal factors (e.g., social functioning) and cognitive function in prostate cancer survivors. In similarly aged populations, greater levels of social stimulation and support have been associated with maintaining cognitive function (Li et al., 2019; Oremus et al., 2019). In prostate cancer, the challenges of managing cancer may strain interpersonal relationships, along with potential subtle cognitive deficits (e.g., word-finding difficulties), and lead to withdrawal or reluctance to engage in social interactions (Ettridge et al., 2018; Wu et al., 2016). Although decreases in social interactions increase the risk of poor cognitive function, the quality of interpersonal relationships can aid with adjustment and coping with prostate cancer (Kamen et al., 2015), which may help maintain cognitive function (Luo et al., 2021). Interestingly, the research on survivors with traumatic brain injuries suggests interpersonal skills training in a neuropsychological intervention can improve both cognitive function and the quality of interpersonal relatedness and interaction (Rattok et al., 1992). Additionally, relationship status may influence the effectiveness and implementation of psychological/behavioral interventions in prostate cancer survivors (Arrato, 2023). Therefore, the relationship between interpersonal factors and cognitive function should be explored in further in prostate cancer survivors undergoing HT especially in the development of psychosocial interventions to improve quality of life outcomes.

### Subjective (Self-Report) and Objective Measures of Cognitive Functioning

Consistent with the evidence base (Crumley et al., 2014; Hutchinson et al., 2012), this review identified a lack of consistency between these measures and their association with psychosocial functioning, lending itself to several explanations. Firstly, unlike objective measures, self-reported cognitive functioning may tap into similar underlying dimensions (e.g., self-perception) as self-reported psychosocial functioning (e.g., DASS-21, POMS). Previous studies have demonstrated strong correlations between self-reported cognitive functioning and depression, anxiety, fatigue, sleep disturbance, and quality of life outcomes in cancer survivors (Hutchinson et al., 2012; Von Ah & Tallman, 2015), though shared method variance (i.e., all these subjective measures are self-reported) may contribute to these findings. A review on self-reported cancer-related cognitive impairment by the Cancer Neuroscience Initiative Working Group (Henneghan et al., 2021) proposed psychological distress should not be dismissed as confounds of self-reported cognitive impairment. They argue, as cognition and distress share similar neural networks and functional implications, self-reported cognitive impairments may be seen as a “separate neural phenotype” of cancer-related cognitive impairment and should be considered part of the clinical picture.

Self-reported measures of cognitive functioning can be valuable in detecting subtle yet pervasive impacts of HT on cognition, which may not be detected on cognitive testing. There are several plausible reasons (Bray et al., 2018). Many traditional tests may be insufficiently sensitive to detect these subtle changes. Testing in ideal conditions (limited distractions and structured one-on-one setting) may also not elicit responses seen in real-world situations where survivors typically experience cognitive problems. Moreover, research participants, who tend to have higher premorbid intellectual functioning compared to the general population, may demonstrate declines to the population average (i.e., high average pre-treatment to average post-treatment). This is consistent with mixed methods research (using neuropsychological testing and qualitative interview) comparing prostate cancer survivors on HT with those not (Wu et al., 2016). Despite a lack of differences on quantitative measures, men on HT reported experiencing more cognitive problems. Although neuropsychological testing remains the “gold-standard” in assessing cognitive function, self-report measures yield important clinical and functional information, which can provide a deeper understanding of the relationship between psychosocial factors and cognitive functions.

### Limitations

The findings of this review must be considered in light of several limitations, which may explain the lack of significant finding on objective cognitive tests. Many studies were low quality (i.e., at high risk of bias), being underpowered, without a clear definition of cognitive impairment, and lacking comprehensive neuropsychological assessment or using cognitive screening only. Regarding the cognitive measures, most studies did not follow ICCTF guidelines on assessing cognitive function, failing to include tests sensitive to cancer-related cognitive impairment let alone sensitive to the effects of androgens (i.e., spatial memory). Most studies also failed to include both a prostate cancer control group (not on HT) and a noncancer control, thereby introducing confounds related to age and the potential interference of cancer itself. In the same vein, some studies did not assess cognition pre-treatment, which may obscure the impact of cancer itself on cognitive (Vardy et al., 2015) and psychosocial functioning. Moreover, many studies were likely subject to selection bias, since men with more severe cognitive problems were either unlikely to participate (answering long questionnaires or neuropsychological testing) or likely to drop out. Finally, not all studies adjusted for practice effects, which may mask subtle cognitive changes signifying decline (Lamar et al., 2003).

### Roadmap for Future Research

Our review identified the following recommendations for future research. Firstly, future studies should adhere to ICCTF recommendations on study design and neuropsychological assessment when evaluating cognitive functions in prostate cancer survivors (Wefel et al., 2011). Ideally, to examine the effect of treatments on cognitions, studies should be double-blinded, randomized, have several control groups (e.g., placebo, prostate cancer-specific, and healthy control groups), prospective, and longitudinal in design. This also includes conducting baseline cognitive assessments before treatment and long-term follow-up and having clearer criteria for cognitive impairment, using neuropsychological measures that have adequate sensitivity to measures affected cognitive domains and psychometric properties, including test-retest reliability, with alternate forms.

Secondly, there is a need to harmonize how psychosocial factors are measured in prostate cancer survivors, especially identifying measures that are sensitive to both age and gender considerations with high reliability and validity for this population. For instance, given that most prostate cancer survivors are older in age, they likely have distinct psychosocial needs compared to younger individuals. Moreover, men may respond differently to certain psychosocial interventions than women (e.g., Zhou et al., 2023). Currently, a lack of consistency pertains across studies regarding the tools and approaches in measurement. Harmonizing these measurement approaches, having established guidelines, will likely aid in identifying and addressing variations to ensure more meaningful and accurate assessments of psychosocial outcomes in prostate cancer survivors.

Thirdly, further research is required to address the knowledge gaps identified in this review with exploring the impact of psychosocial factors beyond psychological distress on cognition. This entails exploring a broader spectrum of factors such as coping behaviors (e.g., self-compassion, mindfulness), interpersonal factors (e.g., social support), the multidimensional aspects of fatigue (e.g., cognitive, emotional), and other factors that may be potential avenues for intervention optimizing cognitive outcomes in prostate cancer survivors.

Finally, the benefit of neuropsychological intervention incorporating strategies to enhance cognition and psychosocial functioning is an area warranting further investigation. While psychosocial interventions exist in the prostate cancer literature, measures of cognitive functions are often not included. Given that cancer-related cognitive impairment is receiving increasing attention as an unmet need in this population, as it is often associated with significant implication on the quality of life, psychosocial, decision-making, financial management, and occupational functioning, including measures of cognitive function in future studies on psychosocial interventions may offer deeper insight into supporting PCS experiencing a myriad of challenges.

## Conclusion

Overall, the research exploring the association between psychosocial factors and cognitive function in prostate cancer survivors undergoing HT is under-developed. While there is some preliminary evidence for associations of psychological distress and fatigue with cognitive function, especially on self-report measures, little is known about the influence of interpersonal factors and coping styles or behaviors. Whether these factors mitigate or exacerbate HT’s effect on cognitive functioning remains to be determined. This information is critical for the optimization of neuropsychological interventions applied in prostate cancer populations.

## Electronic Supplementary Material

Below is the link to the electronic supplementary material.

Supplementary Material 1

## Data Availability

N/A—data is provided in the manuscript along with supplementary materials.
