# Supplementary material for: Psychosocial Factors Associated with Cognitive Function in Prostate Cancer Survivors on Hormonal Treatments: A Systematic Review
Source: Neuropsychol Rev. 2024 Apr 20;35(2):319–36. doi: 10.1007/s11065-024-09639-1 (PMC12328468; doi:10.1007/s11065-024-09639-1)
Supplement: Supplementary file 1 — Supplementary Material 1 [file 11065_2024_9639_MOESM1_ESM.docx]

| **DOMAIN 1: PATIENT SELECTION** | **A. Risk of Bias** | Describe methods of patient selection: |
| --- | --- | --- |
|  |  | Was a consecutive or random sample of patients enrolled? (I.e., whether the participants are representative of the target population) |
|  |  | Was a case-control design avoided? (i.e., cognitive impairment vs no cognitive impairment) |
|  |  | Did the study avoid inappropriate exclusions? |
|  |  | **Could the selection of patients have introduced bias?** |
|  | **B. Concerns regarding applicability** | Describe included patients (prior testing, presentation, intended use of index test and setting): |
|  |  | **Is there concern that the included patients do not match the review question?** |
| **DOMAIN 2: INDEX TEST(S)** | **A. Risk of Bias** | Describe the index test and how it was conducted and interpreted: |
|  |  | Have appropriate tests been used (i.e., not using cognitive screening measures and using neuropsychological measures with adequate psychometric properties)? |
|  |  | Were the index test results interpreted without knowledge of the results of the reference standard? (blind administration) |
|  |  | If a threshold was used, was it pre-specified? (i.e., criteria for cognitive impairment/decline established prior to analysis) |
|  |  | **Could the conduct or interpretation of the index test have introduced bias?** |
|  | **B. Concerns regarding applicability** | **Is there concern that the index test, its conduct, or interpretation differ from the review question?** |
| **DOMAIN 3: REFERENCE STANDARD** | **A. Risk of Bias** | Describe the reference standard and how it was conducted and interpreted: |
|  |  | Is the reference standard likely to correctly classify the target condition? (i.e., is the criteria and the tests used sensitive enough to detect at least mild to moderate cognitive difficulties. Note: ICCTF recommendations and not relying on cognitive screening measures) |
|  |  | **Could the reference standard, its conduct, or its interpretation have introduced bias?** |
|  | **B. Concerns regarding applicability** | **Is there concern that the target condition as defined by the reference standard does not match the review question?** |
| **DOMAIN 4: FLOW AND TIMING** | **A. Risk of Bias** | Describe any patients who did not receive the index test(s) and/or reference standard or |
|  |  | Describe the time interval and any interventions between index test(s) and reference standard: |
|  |  | Was there an appropriate interval between test administration? (i.e., sufficient test-retest intervals according to the literature, or at least adjusting for practice effects for appropriate intervals). |
|  |  | Did all patients receive the same reference standard? |
|  |  | Were all patients included in the analysis OR explanation of dropouts and numbers included for analysis provided? |
|  |  | **Could the patient flow have introduced bias?** |
